# Patient experiences and clinical outcomes of admissions to municipal acute wards versus a hospital: a multicentre randomised controlled trial in Norway

**DOI:** 10.1080/02813432.2024.2377727

**Published:** 2024-07-15

**Authors:** Vivian Nystrøm, Hilde Lurås, Tron Moger, Ann-Chatrin Linqvist Leonardsen

**Affiliations:** a Department of Health, Welfare and Organisation, Østfold University College, Halden, Norway; b Department of Health Management and Health Economics, Norway, University of Oslo, Oslo, Norway; c Health Services Research Unit, Akershus University Hospital, Lørenskog, Norway; d Institute of Clinical Medicine, University of Oslo, Lørenskog, Norway; e Department of Anesthesia, Østfold Hospital Trust, Grålum, Norway

**Keywords:** Health services research, municipal acute ward, primary healthcare, quality indicator, randomised controlled trial

## Abstract

**Background:**

In Norway, municipal acute wards (MAWs) were implemented as alternatives to hospitalisation. Evaluations of the quality of MAW services are lacking. The primary objective of this study was to compare patient experiences after admission to a MAW versus to a hospital. The secondary objective was to compare ‘readmissions’, ‘length of stay’, ‘self-assessed health-related quality of life’ as measured by the EuroQol 5 items 5 level (EQ-5D-5L) index, and ‘health status’ measured by the RAND-12, in patients admitted to a MAW versus a hospital.

**Methods:**

A multicentre randomised controlled trial (RCT), randomising patients to either MAW or hospital.

**Results:**

In total, 164 patients were enrolled in the study; 115 were randomised to MAW and 49 to hospital. There were no significant differences between the MAW and hospital groups regarding patient experience, which was rated positively in both groups. Patients in the MAW group reported significantly better physical health status as measured by the RAND-12 four to six weeks after admittance than those randomised to hospital (physical component summary score, 31.7 versus 27.1, *p* = 0.04). The change in EQ-5D index score from baseline to four to six weeks after admittance was significantly greater among patients randomised to MAWs versus hospitals (0.20 versus 0.02, *p* = 0.03). There were no other significant differences between the MAW and hospital groups.

**Conclusions:**

In this study, patient experiences and readmissions were similar, whether patients were admitted to a MAW or a hospital. The significant differences in health status and quality of life favouring the MAWs suggest that these healthcare services may be better for elderly patients. However, unfortunately we did not reach the planned sample size due to challenges in the data collection posed by the Covid-19 pandemic.

## KEY POINTS

Municipal acute wards have been implemented in Norway as alternatives to hospitalisation. However, the quality of these wards remains unexplored. Results in this study indicates that

- patient experiences after stays in municipal acute wards are equally positive to experiences after stays in hospital
- there are no significant differences in length-of-stay, readmission rates or mortality between municipal acute wards and hospital
- patients have slightly more positive self-rated health and health status 4–6 weeks after staying in a municipal acute ward

## Introduction

A large number of elderly and chronically ill persons are the leading cause underlying the increased pressure on hospitals in many Western countries [1]. Consequently, to utilise healthcare resources in a sustainable way and deliver high-quality services achieving universal health coverage, policymakers worldwide are searching for viable and high-quality healthcare model alternatives [2].

As such, transfer of medical treatment and care from hospitals to primary healthcare has been a key idea in high-income countries [3]. This has led to the development of decentralised acute care units, such as community hospitals units [4]. In Norway, municipal acute wards (MAWs) have been implemented in primary care as alternatives to hospitalisation [5]. Since 2016, all municipalities in Norway have been demanded by law to offer such healthcare services [6]. In 2019, 216 MAWs had been established, with a range from 3 or fewer to 15 or more beds. According to national guidelines, patients admitted to a MAW should have a clarified medical condition or worsening of a known chronic condition, with little risk of deterioration, and the medical treatment should be considered complete within three days. The municipalities are free to organise MAW services according to their own needs [7].

Studies exploring the impact of the implementation of alternatives to hospitalisations are inconclusive: one study shows no reduction in hospitalisations [8], while another reports reductions in early readmission and costs [9]. According to Swanson et al. [10], the implementation of MAWs has led to a 1.9% reduction in hospital admissions for elderly individuals over 80 years. Qualitative studies indicate that patients’ experiences with MAW services are positive and that patients prefer being admitted to a MAW rather than to a hospital [11, 12]. Some studies have been conducted to evaluate the efficacy of the implementation of primary care alternatives to hospitalisation [13], but few of these have assessed the quality of healthcare provided here, versus in hospitals. Only one small randomised controlled trial has been conducted in one MAW in Norway, including in total 60 patients [14]. There were no significant differences in health outcomes between the MAW and hospital, however the authors underlined a need for further research to confirm these findings.

According to the World Health Organisation [15] quality of care is defined as the degree to which healthcare services increase the likelihood of desired health outcomes. This includes that healthcare services should be effective, safe, people-centred, timely, equitable, integrated, and efficient. A recommendation is to use different quality indicators when measuring quality. Quality indicators are used as indirect measures of quality in healthcare [15, 16], and includes for example readmissions, length of stay or incidence of infections. Also, in the era of value-based health care services, patient experience has become a key quality indicator. Patient experiences are positively associated with patient safety and clinical effectiveness [16].

There is a lack of randomised controlled trials exploring the quality of healthcare in primary care alternatives to hospitalisation and in hospital. Also, the organisation of MAWs differs from other primary care alternatives to hospitalisation, and international studies may not be generalisable to a Norwegian setting.

Hence, the aim of the current study was to evaluate whether patients admitted to a MAW receive the same quality of healthcare as those admitted to a hospital. The primary objective was to compare patient experience after admission to a MAW versus to a hospital. The secondary objective was to compare other clinical outcomes, such as readmission, length of stay, self-assessed health-related quality of life, and health status.

## Materials and methods

The study adheres to the Consort 2010 checklist for reporting a randomised trial [17]. The study was carried out in accordance with relevant guidelines and regulations (see ethics approval) and registered at ClinicalTrials.gov, reference number: NCT03885206, first registration 21/03/2019.

### Study design

The study used a randomised controlled trial design (randomising patients assessed by an out-of-hour physician and assumed appropriate for treatment at a MAW to receive treatment either at a MAW or in the hospital).

### Setting and participants

The study was conducted in a non-university hospital’s catchment area in southeastern Norway from September 2019 to January 2021. The area included five MAWs. The MAWs had four to 11 beds, and they were organised as intermunicipal units, co-located with a short-term nursing home. The MAWs were staffed by nurses all day and by physicians during office hours. If the admitting physician requires more diagnostic tests before admission to a MAW, the patients may be sent for extended diagnostics at the hospital first and then transferred to the MAW. When patients admitted to a MAW need further diagnostics, they must be sent to the hospital.

The inclusion criteria were age ≥18 years, ability to provide written informed consent to participate, ability to understand and communicate in Norwegian, and an assessment as appropriate for MAW admission by a primary care physician at the out-of-hour service on the same day. The exclusion criteria were psychiatric diagnosis or cognitive impairment, no registration with a Norwegian social security number, and a previous admission to a MAW.

All patients considered appropriate for admission to a MAW but sent to the hospital for extended diagnostics were included.

### Recruitment, randomisation and blinding

A study nurse recruited patients assessed as appropriate for MAW admission by an out-of-hour physician at one of the five out-of-hour services in the catchment area. Participants were randomly assigned to either a MAW or the hospital at a 2:1 allocation, to alleviate the pressure on the hospital. The randomisation was computer-generated and stratified with a block size of 100 for each of the five out-of-hour services. Hence, there were 100 sealed pre-randomized envelopes in each out-of-hour service. If the patient consented to participate, the study nurse drew a sealed envelope with a unique study number and information about where the patient was allocated. Due to the nature of the study, blinding for the intervention was not possible.

### Primary outcome

The primary outcome was patient experiences four to six weeks after admittance to a MAW or to the hospital as measured by The Nordic Patient Experience Questionnaire (NORPEQ) [18]. The NORPEQ has been validated and tested for reliability and consists of eight items. Six items concern experiences with health personnel, including: whether the doctors were understandable, doctors’ and nurses’ professional skills, nursing care, whether the doctors and nurses were interested in the patient’s problems, and information relating to tests. Further, two items relate to general satisfaction and incorrect treatment. All items use a five-point descriptive scale with the response categories: ‘not at all’, ‘to a small extent’, ‘to a moderate extent’, ‘to a large extent’, and ‘to a very large extent’. The first six items may be summed to an overall score, and calculated by adding the responses for the six items and transforming the scores to a 0–100 scale where 100 represents the best possible experience of care. Values in the NORPEQ questionnaire was transformed as followed: value 1 was transformed to value 20, value 2 was transformed to value 40, value 3 was transformed to value 60, value 4 was transformed to value 80 and value 5 was transformed to value 100.

### Secondary outcomes

The secondary outcomes were 30-day readmission rate; 30-day mortality rate; length of stay; health-related quality of life measured by the EQ-5D-5L (European Quality of Life 5 dimensions, 5 levels questionnaire) [19] at baseline and four to six weeks after admission; and patient-reported health status measured by the RAND-12 (20) four to six weeks after admission.

The EQ-5D-5L questionnaire includes five questions covering mobility, self-care, usual activities, pain/discomfort, and anxiety/depression and the visual analogue scale (EQ VAS). The responses were on a 5-point Likert scale, where 1= ‘not at all’, 2= ‘to a small extent’, 3= ‘to a moderate extent’, 4= ‘to a large extent’ and 5= ‘to a very large extent’. The EQ-5D-5L index score was aggregated according to the Norwegian Medicines Agency, which recommends using the UK EQ-5D-3L value set [19]. The EQ-5D index utility score ranges from 0 to 1, where 0 indicates a worse health scenario and 1 indicates the best health scenario. The EQ VAS ranges from 0 to 100, where 0 represents the worst possible self-assessed health-related quality of life and 100 the best possible.

The RAND-12 (20) consists of 12 questions covering general health, physical functioning, performance of responsible tasks, body pain, mental health, energy/fatigue and social functioning. These 12 questions may be aggregated into a physical health summary scale component (PCS) and a mental health summary scale component (MCS), with score values ranging from minimum ‘0’ to maximum ‘100’.

The 30-day readmission rate and mortality rate were registered 30 days after discharge and collected from the patients’ medical journal. Length of stay was calculated as the discharge date minus the admission date.

### Sociodemographic variables

The following variables were collected at the out-of-hour service at baseline: gender, age, International Classification of Primary Care (ICPC)-2 diagnosis main diagnosis group, and the National Early Warning Score (NEWS). NEWS is based on six clinical values: respiratory rate, oxygen saturation, systolic blood pressure, pulse/heart rate, level of consciousness and body temperature [21].

The Charlson comorbidity index (CCI) [22], which classifies comorbid conditions that might alter the risk of mortality, was scored based on information collected from the patients’ medical records. The CCI measures 19 conditions that are weighted individually according to their strength, from a minimum of ‘0’ points to a maximum of ‘37’ points.

### Sample size calculation

We assumed a type I error of 0.05% and 80% power based on the NORPEQ scores in a recent MAW study [23]. The mean NORPEQ score in MAWs was 83 with a standard deviation (SD) of 13. Previous data for a sample of patients from the same hospital indicated a NORPEQ score of 78 [24]. Hence, a result showing superiority (25) for patients admitted to MAWs was expected. Following a superiority design, 2:1 allocation to the MAWs and hospital respectively, the required sample size was 400 participants in total, 270 in MAWs and 130 in hospitals. The 2:1 allocation was done to reduce the burden of additional patients for the hospital. Adjusted for potential drop-outs (5% dead, 15% non-response to follow-up) an inclusion of 340 patients in MAWs and 160 patients in hospital was planned. The mortality rate was based on observations from one of the largest MAWs in the county, and the drop-out rate was assumed similar to previous studies [23].

### Data collection

At baseline, study nurses at all five out-of-hour services registered each patient’s sociodemographic variables, NEWS score and whether the patient was sent to the hospital for extended diagnostics before being transferred to a MAW. After consenting to participate, the patient completed the EQ-5D-5L questionnaire. The patient received the questionnaires (EQ-5D-5L, RAND-12 and NORPEQ) on paper and was instructed to return these questionnaires by post to the first author in a pre-stamped envelope after four weeks. If the questionnaires were not received within six weeks, the first author called the patient, and the questionnaires were completed during a telephone interview.

The discharge date, mortality within 30 days of discharge (all causes), whether the patient was readmitted to the hospital or a MAW within 30 days (all causes), and information needed to score the CCI were collected from the patients’ medical record in either the MAW or hospital by the first author.

### Analyses

Descriptive statistics are presented as numbers and percentages, the mean with standard deviation (SD) or the median and interquartile range (IQR), as appropriate. To test for differences in 30-day readmission rate and 30-day mortality rate between the MAW and hospital groups, chi-square tests were performed. T-tests were performed to assess differences between the groups in the length of stay, EQ-5D index after 4 weeks, change in EQ-5D between 4 weeks and baseline, and summary score of PCS and MCS. The Mann–Whitney U test was employed to assess the NORPEQ variables.

The intention to treat analysis are presented by tables in this paper. A significance level of 5% (two-sided) was used throughout. All analyses were performed with IBM Statistical Package for the Social Sciences (SPSS) version 27.

## Results

**Figure 1.**
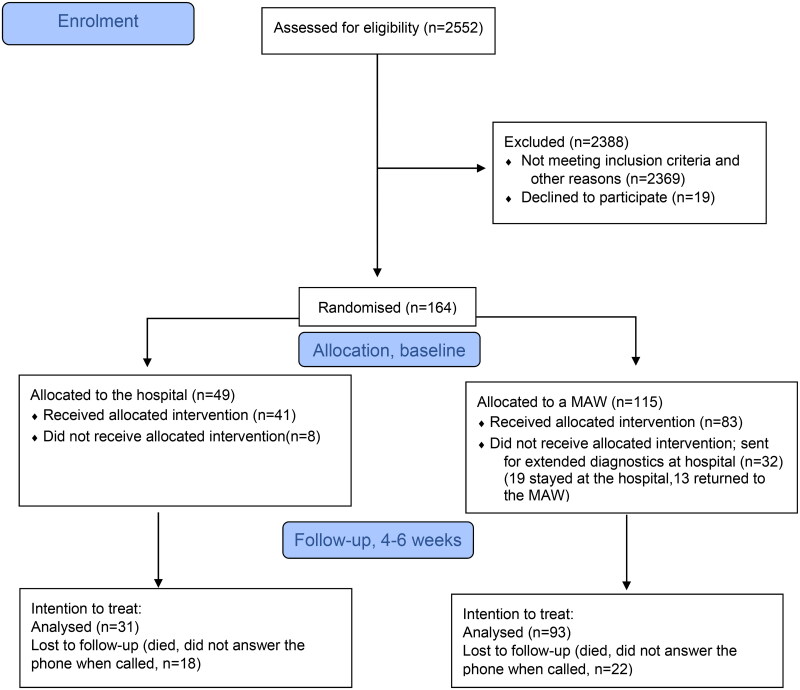
CONSORT 2010 flow diagram.

Patients were recruited from September 2019 to January 2021. The study was paused during the period from March 12th, 2020, to June 1st, 2020, due to the COVID-19 pandemic and extensive pressure on the hospitals’ emergency departments. In total, 164 patients were enrolled and randomized in the study; 115 were randomised to a MAW and 49 to the hospital. Study recruitment, losses and exclusions are summarised in the CONSORT flow diagram (Figure 1).

### Participant characteristics

Table 1 presents the baseline characteristics (*n* = 164).

**Table 1.** Participant gender, age, ICPC-2 main group, CCI, EQ-5D index and NEWS at inclusion = 164.

	Total N = 164 n (%)	MAW *N* = 115 n (%)	Hospital *N* = 49 n (%)
Female	106(64.6)	77(67.0)	29(59.2)
Male	58(35.4)	38(33.0)	20(40.8)
Age (mean/SD)	69.6(20.4)	70.7(20.6)	66.9(19.9)
**ICPC2 main group**			
General and unspecified	18(11.0)	14(12.2)	4(8.2)
Digestive	24(14.6)	16(13.9)	8(16.3)
Ear	2(1.2)	2(1.7)	0(0)
Cardiovascular	3(1.8)	2(1.7)	1(2.0)
Musculoskeletal	42(25.6)	30(26.1)	12(24.5)
Neurological	8(4.9)	7(6.1)	1(2.0)
Psychological	1(0.6)	1(0.9)	0(0)
Respiratory	37(22.6)	23(20.0)	14(28.6)
Skin	4(2.4)	4(3.5)	0(0)
Endocrine/metabolic and nutritional	7(4.3)	4(3.5)	3(6.1)
Urological	13(7.9)	10(8.7)	3(6.1)
Pregnancy, childbearing, family planning	3(1.8)	1(0.9)	2(4.1)
	**Mean (SD)**	**Mean (SD)**	**Mean (SD)**
CCI	4.5(3.0)	4.4(2.9)	4.6(3.3)
EQ-5D index	0.30(0.4)	0.30(0.4)	0.30(0.4)
NEWS	2.5(2.4)	2.4(2.4)	2.8(2.3)

MAW = Municipal acute ward. SD = standard deviation. ICPC2 main groups = The International Classification of Primary Care-2, equal to the reason for admittance. CCI = Charlson comorbidity index score. EQ-5D index = utility score ranging from 0 to 1, 0 indicates the worst health scenario and 1 indicates the best health scenario. NEWS = National Early Warning Score. Intention-to-treat analysis.

In our sample, there were indications that more women and slightly younger patients were allocated to the hospital. However, the differences were small.

### Primary outcome

Table 2 presents patient experiences after admission as measured by the NORPEQ.

**Table 2.** Patient experience 4–6 weeks after admittance (T2) to MAW versus hospital, NORPEQ, *n* = 124.

	MAW *n* = 93				Hospital *n* = 31				p value
NORPEQ total score* Mean (SD) Median (IQR)	88(12.4) 93(80–100)				86 (17.9) 93 (80–100)				0.93
	Mean (SD)	Small n (%)	Moderate n (%)	Large n (%)	Mean (SD)	Small n (%)	Moderate n (%)	Large n (%)	
Doctors were understandable	4.4(0.8)	3(3.2)	8(8.6)	82(88.2)	4.3(1.2)	3(9.7)	2(6.5)	26(83.9)	0.85
Doctors’ professional skills	4.4(0.9)	5(5.3)	8(8.5)	81(86.2)	4.5(1.1)	2(6.5)	1(3.2)	28(90.3)	0.74
Nurses’ professional skills	4.6(0.7)	1(1.2)	3(3.2)	89(95.7)	4.6(0.9)	1(3.2)	2(6.5)	28(90.3)	0.72
Nursing care	4.5(0.7)	2(2.3)	4(4.3)	88(93.6)	4.6(0.7)	0(0)	4(12.9)	27(87.1)	0.85
Doctors and nurses interested in problem	4.3(0.9)	2(2.2)	17(18.5)	73(90.8)	4.0(1.3)	3(9.7)	8(25.8)	20(64.5)	0.53
Information on tests	4.1(1.2)	10(11.1)	11(12.2)	69(76.7)	3.8(1.7)	7(22.6)	2(6.5)	22(71.0)	0.81
General satisfaction	4.5(0.9)	4(4.3)	7(7.5)	83(88.3)	4.2(1.4)	4(12.9)	2(6.5)	25(80.6)	0.44
Incorrect treatment	1.3(0.9)	83(89.2)	5(5.4)	5(5.4)	1.6(1.3)	27(87.1)	0(0)	4(12.9)	0.60

T2= questionnaire tine 2 – 4–6 weeks after admittance. MAW = Municipal acute ward. IQR = interquartile range. SD = standard deviation.

* The NORPEQ total score is scored 0–100; 100 represents the best possible patient experiences All items use five-point descriptive scales with the response categories 1=’not at all’, 2=’to a small extent’, which were collated to 1=’to a small extent’ (=Small), 3=’to a moderate extent’ (=Moderate) was converted to the value 2, 4=’to a large extent’ and 5= ‘to a very large extent’ were collated to 3=’to a large extent’ (=Large). Ho; there is no difference in patient experiences between the MAW and the hospital, measured with the Mann–Whitney U test. The p value was assumed significant at the 0.05 level. Intention-to-treat analysis.

The total mean score was slightly in favour of MAW (88 vs. 86). There were no significant differences in patient experiences between the MAWs and the hospital.

### Secondary outcomes

Table 3 gives an overview of the secondary outcomes after randomisation to a MAW versus the hospital.

**Table 3.** Secondary outcomes at admittance to a MAW versus a hospital, *N* = 164.

	Total *N* = 164 n (%)	MAW *N* = 115 n (%)	Hospital *N* = 49 n (%)	P value
30-day readmission	43(26.2)	31(27.0)	12(24.5)	0.85
30-day mortality	9(5.5)	4(3.5)	5(10.2)	0.08
	**Mean (SD)**	**Mean (SD)**	**Mean (SD)**	
Length of stay	3.4(2.8)	3.6(2.5)	2.8(3.3)	0.10
EQ-5D-5L	0.50(0.4)	0.50(0.3)	0.40(0.4)	0.02*
EQ-5D-5L difference	0.20(0.4)	0.20(0.4)	0.02(0.4)	0.03*
PCS-12	30.5(10.8)	31.7(10.8)	27.1(10.3)	0.04*
MCS-12	48.9(13.1)	48.3(13.4)	50.7(12.3)	0.38

MAW = Municipal acute ward. 30-day readmission = patients readmitted to either a MAW or the hospital within 30 days, all causes. 30-day mortality = patient died within 30 days, all causes. EQ-5D-5L = European Quality of Life, 5 dimensions, 5 level questionnaire; presented as EQ-5D index = utility score ranging from 0 to 1, where 0 indicates the worst health scenario and 1 indicates the best health scenario, 4–6 weeks after admittance. EQ-5D-5L difference = the difference between the EQ-5D index score at baseline and that at 4–6 weeks after admittance. PCS-12 = physical component summary score 4–6 weeks after admittance, MCS-12 = mental component summary score 4–6 weeks after admittance, as measured with the RAND-12. SD = standard deviation. Ho; there is no difference in 30-day readmission and 30-day mortality between the MAW and the hospital, measured with the Chi-square test. Ho; there is no difference in length of stay, EQ-5D-5L 4–6 weeks after admittance, EQ-5D-5L difference, PCS-12, or MCS-12 between the MAW and the hospital, measured with a T test. *P value significant at the 0.05 level. Intention-to-treat analysis.

Patients in the MAW group (*n* = 93) reported significantly better physical health on PCS-12 after four to six weeks than those randomised to hospital (*n* = 31), 31.7 versus 27.1 (*p* = 0.04). EQ-5D index score after four to six weeks were significantly higher in the MAW group (*n* = 93) than in the hospital group (*n* = 31), 0.50 versus 0.40 (*p* = 0.02). The change in EQ-5D index score from baseline to four to six weeks after admittance was greater for patients randomised to a MAW than for patients randomised to the hospital. The mean change score was 0.20 at the MAW and 0.02 at the hospital (*p* = 0.03).

## Discussion

To our knowledge, this is the first multicentre RCT comparing the healthcare quality in MAWs versus a hospital. The only significant differences between the MAWs and the hospital were in the patients’ self-assessed health-related quality of life and in the self-rated health four to six weeks after admission, both in favour of MAWs. There were several non-significant differences regarding both patient experiences and patient outcomes.

### Strengths and limitations

We aimed to compare quality measures within two healthcare services, and a strength of the study was the RCT design. The recruitment process was extensive as patients were randomly selected from five out-of-hour services and then assigned to one of five MAWs or to hospital. In addition, data were collected from several sources, such as questionnaires, telephone interviews and medical records, which made the dataset complete. However, patients are referred to MAWs also from general practitioners and nursing home. This may limit the external validity of the study.

A challenge with this RCT is that the included sample is limited to consent-competent individuals. This may represent a bias in the results, and may also limit the external validity. The MAWs in Norway vary in range and size, which might affect the generalizability. Thus, the study does not necessarily have high external validity.

We did not achieve the estimated sample size. Due to the small study population, we acknowledge that the study suffers from a substantial risk of type 2 error when testing for differences in both the primary and secondary outcomes. Detecting differences in mortality and readmissions in feasible sample sizes was considered difficult already during study planning, due to the low mortality rate and small expected differences in both outcomes. Nevertheless, the study is larger than previous trials on MAWs in Norway. Not reaching the desired sample size was mainly due to the COVID-19 pandemic, which put pressure on the hospital. Other reasons not to be enrolled in the randomization were that the patient did not want to be admitted to the hospital, that there were no available beds at the MAW, or that the physicians considered it to be unethical to send the patient to the hospital when he or she could be treated at the local MAW.

In addition, asking patients to report their experiences after 4–6 weeks may represent a risk for recall bias.

### Study findings in relation to previous research

Overall, patient experience was rated positively regardless of allocation. This corresponds with results from a Swedish pilot RCT that found that patients allocated to the Community Health Centre (*n* = 105) did not experience lower levels of trust and patient safety compared to patients referred to the hospital (*n* = 83) [26]. Even if the difference was not significant, patients allocated to a MAW reported that health personnel were more interested in how they described their own situation and with the information they received during their stay than patients allocated to the hospital. This is in line with findings from a study showing that patients find it difficult to contact and obtain information from hospital physicians [27]. Another study highlights that the way healthcare personnel communicate and provide information about a patient’s health condition is crucial for whether patients experience being met with warmth and respect [28]. This is also in-line with findings from a Norwegian RCT comparing healthcare services at a community hospital with four MAW beds and a hospital, where patients admitted to the community hospital (*n* = 33) reported higher satisfaction than those admitted to the hospital (*n* = 27) [11].

After four to six weeks, patients allocated to the hospital reported lower health-related quality of life than patients allocated to a MAW. The MAW is reported to have a calmer environment than the hospital, making it easier to provide holistic care [23]. Thus, incorporating patient empowerment into treatment, the patients can be prepared to handle their condition at home. Also, it may be more feasible collaborating within the same healthcare level, facilitating the transfer from MAW to home care services.

In our sample, we observed a difference in the 30-day mortality rate of 3.5% in the MAWs and 10.2% in hospital. Also, Garåsen et al. [29] found that treatment in a community hospital (*n* = 72) led to a lower mortality than hospital treatment (*n* = 70). Hilland et al. [30] found that the implementation of MAWs was associated with a reduction in both mortality and readmission rates. Likely due to the inadequate sample size, these differences were not statistically significant in our study. However, it may be argued that these differences are clinically significant.

In the study from Lappegaard et al. [14] the readmission rate was higher than both in the hospital and in the MAWs in our study. Other studies have shown that continuity of care reduces the readmission rate to hospitalsT [31]. Actions such as discharge planning, aiming for better collaboration between healthcare levels to improve patient pathways, have been shown to be effective in reducing readmissions [32]. This may indicate quality and continuity of care in both the hospital and MAWs in our study.

## Conclusion

This study underlines the challenges of conducting multicentre RCTs across hospital and primary care. However, based on the findings in our sample, we suggest that MAWs offer equally good quality compared to hospitals. Further studies should aim to explore which patients benefit most from receiving treatment in units such as MAWs. Further implications for clinics are to prepare criteria for the identification and selection of the right patients for MAWs.

## Declarations

## Data Availability

Datasets generated and/or analysed during the current study are not publicly available due to local ownership of data, but aggregated data are available from the corresponding author on reasonable request.
